# Opportunities and Limits in Salvage Surgery in Persistent or Recurrent Head and Neck Squamous Cell Carcinoma

**DOI:** 10.3390/cancers13102457

**Published:** 2021-05-18

**Authors:** Gerhard Frank Huber

**Affiliations:** 1 HNO-Klinik, Kantonsspital St. Gallen, Rorschacher Strasse 95, 9007 St. Gallen, Switzerland; gerhard.huber@kssg.ch or huber@orl-zentrum.com; 2 Zentrum für Ohren-, Nasen-, Hals- und Plastische Gesichtschirurgie, Klinik Hirslanden, Witellikerstrasse 40, 8032 Zürich, Switzerland

**Keywords:** head and neck cancer, cancer recurrence, cancer persistence, squamous cell carcinoma, treatment failure, salvage surgery

## Abstract

Except for HPV-induced cancers of the oropharynx, survival rates in patients with squamous cell carcinoma of the head and neck (HNSCC) have not changed substantially over the last decades. Salvage surgery plays an important role where primary treatment was unsuccessful since 50% of advanced-stage patients relapse after nonsurgical primary treatment. Depending on a variety of factors, a considerable number of patients in whom primary treatment was not successful can still be cured by salvage surgery. It is the goal of this review to elucidate these factors with the aim to counsel patients and their relatives realistically about the chances of being cured.

## 1. Introduction

For the last 20–30 years, treatment of primary Head and Neck Cancers by radiotherapy (RT) or chemoradiation (CRT) has constantly been increasing and became a gold standard in many HNSCC. While in the 1960s, primary surgical treatment of laryngeal cancer was done in >90% of cases, it has decreased to ~50% nowadays. This is among other reasons attributable to landmark studies, such as the “VA-Study”, that offered comparable survival rates and a reasonably high rate of organ preservation in patients treated by induction chemotherapy and RT compared to patients treated primarily with surgery and adjuvant RT [1]. Further, for some HNSCC in certain subsites it has been shown that primary RT should be the standard of care or at least the most preferable treatment modality (nasopharyngeal cancers, hypopharyngeal tumors, respectively). On the other hand, SCC of the oral cavity or the skin is primarily treated by surgical approaches because of its favorable outcomes [2]. Nevertheless, the majority of HNSCC disease at Stage III or IV is primarily treated by a combination of surgery and radiotherapy (multimodal treatment).

Despite aggressive RT or CRT, about 50% of patients experience relapse [3]. Persistent cancer is rather imprecisely defined as a lack of complete remission of a tumor in its primary site or regional lymph nodes within the initial “few months” after treatment termination (unfortunately, there is no uniform agreement to discriminate between persistence and recurrence). Recurrence, therefore, would be the regrowth of a tumor in its primary site or regional lymph nodes when a complete remission had been documented after treatment completion.

Persisting and recurrent disease have to be discriminated from a second primary since in the latter, the outcome is known to be better.

Persistent/recurrent disease poses several challenges and, therefore, should be assessed systematically by a team experienced in the whole range of management options available, including surgical salvage, re-irradiation, chemotherapy, and palliative care [4] and a thorough selection of patients appropriate for a surgical procedure is key.

## 2. Challenges to Be Encountered

### 2.1. First Challenge: Timely Detection of Recurrent Disease

The smaller the recurrence, the better the chances of salvage.

Although under normal circumstances, HN patients are integrated in an orderly follow-up some patients will not show up on a regular basis or are even lost for follow-up. Detection of a recurrent tumor is challenging due to several factors: in patients primarily treated by radiotherapy, tumor cells often survive close to hypoxic areas in the center of the previous tumor or lymph nodes and not on the surface. In addition, in the case of initial flap reconstructions, a recurrence on the primary site is likely to be hidden/buried by the free flap.

Due to its high negative predictive value, PET scan is an excellent tool to discriminate radiation-induced edema (PET-negative) from a recurrent tumor (PET-positive) [5] and, therefore, may prevent patients from unnecessary biopsies. In the case of laryngeal cancers, unnecessary post-biopsy swelling and consecutive tracheostomy can hence be avoided.

### 2.2. Second Challenge: Exclusion of Distant Disease

If recurrence/persistence is confirmed by fine-needle aspiration cytology (FNAC) or open biopsy, the next step is to preclude distant disease: Again, a PET scan is an excellent tool, as is a triple endoscopy combined with a CT Scan of thorax, abdomen. If there is suspicion of distant disease by a PET or CT scan, in most cases, biopsy or FNAC are warranted. Distant disease does not always exclude curative treatment or predetermine a purely palliative setting: in oligo-metastatic disease, solitary nodules in the lung can still be addressed by surgery (wedge resection or pneumonectomy) or stereotactic radiation, as can solitary metastases in the vertebral column or elsewhere.

### 2.3. Third Challenge: Assessment of Tumor Extension

As a general rule: the bigger the recurrence, the lower the chances of successful salvage.

In oligo-metastatic or absent distant disease, the size of the recurrence has, therefore, to be assessed; this is somewhat difficult through the tissue changes after previous radiotherapy and/or surgery. The discrimination between inflammatory changes, scar tissue formation, and recurrent disease is not easily possible, even in the hands of experienced H&N radiologists [6]. Very often, the combination of PET scan [7] and MRI can provide sufficient information about the expected tumor dimensions and involvement of nearby crucial structures, such as the internal carotid artery, which leads to the next challenge.

### 2.4. Fourth Challenge: Assessment of Resectability

The involvement of the prevertebral fascia, common or internal carotid artery render a tumor inoperable by definition (rcT**4b**). Further, the rare involvement of the cervical or brachial plexus and upper esophageal sphincter limits the options of salvage surgery. It has to be underlined that the decision of whether a tumor is resectable or not has to be made on an individual basis. Even though it has been shown by earlier studies that patients who underwent resection of the carotid artery did not have improved survival compared to patients in a palliative setting, it is important that at least the goal of loco-regional tumor control was achieved in the majority of patients, which, compared to unbridled tumor growth, is already a remarkable success [8].

In the case of a tumor that has been judged as resectable, it is of importance to counsel the patient about his/her chances of successful surgery. Several factors have been found to influence the success rate of salvage surgery: rcT stadium, rcN stadium, simultaneous oligo-metastatic disease, persistence vs. recurrence, and in the latter, time elapsed before recurrence diagnosis, regional and/or local recurrence with an early recurrence as a negative predictor of outcome [9]. Nevertheless, it is noteworthy that not only the cure rate is of importance but also the survival, as shown by a recent study by Patil VM et al. [10], where patients who underwent salvage neck dissection survived substantially longer than the palliative control group (22 months vs. 9.7 months; *p* = 0.000).

## 3. Predictors of Success

### 3.1. Site of Recurrence

#### 3.1.1. Primary Tumor Site

Depending on the site of recurrence, salvage surgery is more or less challenging. Different subsites show different chances of successful salvage, and this has to be considered in treatment planning [11].

##### Nasopharynx

A tumor recurring in the nasopharynx is difficult to salvage. Even though experienced rhinosurgeons can easily access this region, a recurrent tumor is mostly ill-defined and close to important structures of the skull base (optic nerves, internal carotid arteries, cavernous sinus), which often prevents complete/radical tumor resection. Furthermore, open access procedures, such as the maxillary swing or similar mid-facial approaches, are only rarely performed in western countries where the incidence of this tumor entity is low compared to East Asia, and thus high patient volume is lacking. With varying degrees of naso-pharyngectomy, cure rates in selected patients have been reported in the region of 40% at five years [4]. Here, as a feasible alternative, re-irradiation, has to be considered.

##### Larynx

A recurrence in the larynx has the propensity to be detected early due to the symptoms, such as hoarseness and aspiration. Therefore, persistent or recurrent disease is often still confined within the larynx, and nodal spread occurs less commonly than in other primary sites due to the sparse lymphatic drainage. Therefore, salvage surgery of laryngeal SCC is generally more successful than in other locations (>70%) [12,13]. Even in the case of recurrent disease, partial surgical approaches still can be considered: partial open (CHEP) vs. endoscopic procedures (laser, TORS). As an ultima ratio, total laryngectomy is the salvage procedure of choice with good functional results. In this case, some studies found the application of a salivary bypass tube and/or a muscular pectoralis major flap for suture line reinforcement to reduce fistula rates noticeably [4].

##### Oropharynx

Most likely due to more aggressive reconstruction techniques and robotic surgery, the revival of primary surgical treatment or at least multimodal treatment, as well as HPV infection as an etiological factor, the outcome in this tumor site has improved over the last two decades. However, due to the proximity to the parapharyngeal space and internal carotid artery and more difficult accessibility compared to the oral cavity, this location of recurrence makes salvage surgery still difficult. While older published survival data were disillusioning [14,15], newer ones show more promising outcomes [16].

##### Hypopharynx

If detected early at first diagnosis, small tumors of the hypopharynx can be treated surgically. The overwhelming majority, however, is treated by a regimen including radiotherapy since the likelihood of laryngeal involvement at the same time is reasonably high, and surgical treatment would often mean total laryngopharyngectomy [17]. Due to its somewhat hidden location, recurrent disease is often detected late, and salvage rates still are (very) low.

##### Oral Cavity

Although recurrent disease can be detected earlier compared to other subsites and a considerable number of patients are initially treated only surgically, even with the possibility of available adjuvant RT or CRT, success rates are worse than in laryngeal cancer. This has sometimes been explained by excessive field cancerization, which is not really a satisfactory explanation, and, therefore, it has not been completely understood so far.

#### 3.1.2. Regional Recurrence

As discussed in Section 2.2., salvage surgery is possible as long as no distant disseminated disease exists. As mentioned, it has to be underlined that even in oligo-metastatic disease, a cure is still possible, and single (-triple) lung metastases can be addressed with wedge resection(s) or stereotactic radiotherapy.

If disease persists (viable tumor cells) in regional lymph nodes, salvage neck dissection is indicated. Treatment with radiotherapy alone, human papillomavirus negative cancers, and an increase in lymph node size, were found to be predictors for viable tumor cells and reduced survival. In case of decreased or stable lymph node size after chemoradiation, watchful waiting can be considered [18].

It is of pivotal importance that in the case of salvage neck dissection, persistence/recurrence is limited to the regional lymph nodes, and the primary site is free of tumor. Otherwise, if a neck dissection is performed and soon thereafter a primary recurrence is detected, reconstruction with free flaps can be compromised (donor vessels of the neck), and tumor seeding can occur in unexpected locations. Therefore, a comprehensive scanning preoperatively for distant and local persistence/recurrence is mandatory.

### 3.2. Time Point of Recurrence/Disease Free Interval on Outcome

If a cancer persists (no evidence that the tumor ever disappeared) or recurs early after treatment, the tumor is obviously resilient and aggressive and has per se a worse outcome than a tumor recurrence, e.g., 4 years after treatment [19,20,21].

### 3.3. Impact of Initial Tumor Extent

Not unexpectedly, numerous studies, such as the one from Agra et al. [22], have demonstrated that initially small primaries (T1,2) and low tumor stage (I,II) show a significantly lower risk of (1) recurrence, (2) distant metastasis, and (3) high rates of success for salvage surgery, and, therefore, a better outcome.

### 3.4. Complication Rates

Salvage surgery has a higher complication rate compared to primary surgical treatment (29–60%) [23,24,25,26,27,28,29], and surgical complications have been found to be an independent risk factor of poor outcome [30]. This has to be considered in the tumor board where patients are discussed upfront, especially in cases where the failure of primary chemoradiation is more likely (e.g., T4a laryngeal or hypopharyngeal cancers, or neck disease with extra nodal extension). Therefore, where future salvage surgery is anticipated to be challenging or impossible, primary surgical treatment should be considered favorably as the first/initial approach.

If salvage surgery is considered, preoperative factors, such as nutrition, thyroid hormone levels, blood glucose control, smoking, and alcohol abuse, can be optimized prior to surgery. In addition, immunonutrition appears to have some positive effect on complication rates and on the duration of hospital stay [31].

## 4. Salvage by TORS

TORS has been found to have some advantages in the resection of oropharyngeal and supraglottic tumors. However, when it comes to salvage surgery, for example, in the tongue base, haptic feedback is lacking, and very often, the tumor is ill-defined. Furthermore, when the recurrent tumor is close to the internal carotid artery, dissection by TORS can end up in uncontrollable bleeding and fatal exit. Therefore, in order to achieve good results, salvage surgery by TORS should be restricted to small, clearly discernable tumors [32], while in other cases, open surgery with direct visual and haptic control is to be preferred.

## 5. Extended Resections

Occasionally, recurrent disease infiltrates the prevertebral fascia or the carotid artery (common, internal) and is therefore judged inoperable (rcT**4b**). Nevertheless, in thoroughly selected patients, salvage surgery is still possible: Although there are several studies suggesting carotid artery resection and reconstruction has no impact on survival, it has to be underlined that locoregional tumor control can be achieved in many cases, and uncontrollable tumor growth in the neck and face with its horrifying consequences for the patients and their families can be prevented.

## 6. Late “Recurrences”

A remarkable number of tumors do not recur within the 5 years of follow-up, and patients are considered cured. However, a certain percentage of patients that have been successfully treated by radiotherapy develop a SCC or sarcoma in the primary location years or even decades after the termination of follow-up and those are regarded as radiation-induced. This poses several challenges: Since those patients are not under medical follow-up anymore, it is likely that they present in a more advanced tumor stage. Due to the previous treatment (either primary or adjuvant Radio[chemo]therapy), these patients are poor candidates for curative re-irradiation. Surgery itself is difficult due to the advanced tumor stage, scar tissue, and in the case of sarcoma, the ill-defined pseudo capsule [33].

## 7. Discussion

A crucial consideration in salvage surgery is an accurate selection of patients suitable for surgery and the realistic counseling of patients regarding their chances of successful treatment. Given the fact that in most cases, adjuvant radiotherapy is not possible due to wide local excision, the salvage resection defect is often considerable, and free flap reconstruction is warranted. This has an impact on function, and not every patient is willing to accept a life without a larynx, tongue, or the inability of oral food intake.

Chances of cure, functional and esthetic deficits have to be discussed honestly, and in case that a patient refuses surgery, he/she has to be advised about a possible course of the disease in the case of palliative treatment or best supportive care. It should be emphasized that not a single person, but a team experienced in the whole range of management options available for recurrent disease, including surgical salvage, re-irradiation, chemotherapy, and palliative care, should be involved in decision making (tumor board).

Technical feasibility must be balanced with the morbidity of a procedure, the functional consequences of organ mutilation, and the likelihood of salvage success.

## References

[1] Wolf G.T., Fisher S.G., Hong W.K., Hillman R., Spaulding M., Laramore G.E., Endicott J.W., McClatchey K., Henderson W.G. (1991). Induction chemotherapy plus radiation compared with surgery plus radiation in patients with advanced laryngeal cancer. Department of Veterans Affairs Laryngeal Cancer Study Group. N. Engl. J. Med..

[2] Studer G., Zwahlen R.A., Graetz K.W., Davis B.J., Glanzmann C. (2007). IMRT in oral cavity cancer. Radiat. Oncol..

[3] Grégoire V., Lefebvre J.L., Licitra L., Felip E. (2010). EHNS-ESMO-ESTRO Guidelines Working Group. Squamous cell carcinoma of the head and neck: EHNS–ESMO–ESTRO Clinical practice guidelines for diagnosis, treatment and follow-up. Ann. Oncol..

[4] Mehanna H., Kong A., Ahmed S.K. (2016). Recurrent head and neck cancer: United Kingdom National Multidisciplinary Guidelines. J. Laryngol. Otol..

[5] Gupta T., Master Z., Kannan S., Agarwal J.P., Ghsoh-Laskar S., Rangarajan V., Murthy V., Budrukkar A. (2011). Diagnostic performance of post-treatment FDG PET or FDG PET/CT imaging in head and neck cancer: A systematic review and meta-analysis. Eur. J. Nucl. Med. Mol. Imaging.

[6] Anderson C.M., Chang T., Graham M., Marquardt M.D., Button A., Smith B.J., Menda Y., Sun W., Pagedar N.A., Buatti J. (2015). Change of SUVmax Slope in Dynamic Triphasic FDG-PET/CT Distinguishes Malignancy from Post-Radiation Inflammation in Head and Neck Squamous Cell Carcinoma—A Prospective Trial. Int. J. Radiat. Oncol. Biol. Phys..

[7] Gao S., Li S., Yang X., Tang Q. (2014). 18FDG PET-CT for distant metastases in patients with recurrent head and neck cancer after definitive treatment. A meta-analysis. Oral Oncol..

[8] Illuminati G., Schneider G., Minni A., Calio F.G., Pizzardi G., Ricco J.B. (2016). Resection of recurrent neck cancer with carotid artery replacement. J. Vasc. Surg..

[9] Zafereo M. (2014). Surgical salvage of recurrent cancer of the head and neck. Curr. Oncol. Rep..

[10] Patil V.M., Noronha V., Thiagarajan S., Joshi A., Chandrasekharan A., Talreja V., Agarwal J., Ghosh-Laskar S., Budrukkar A., Juvekar S. (2020). Salvage surgery in head and neck cancer: Does it improve outcomes?. Eur. J. Surg. Oncol..

[11] Matoscevic K., Graf N., Pezier T.F., Huber G.F. (2014). Success of salvage treatment: A critical appraisal of salvage rates for different subsites of HNSCC. Otolaryngol. Head Neck Surg..

[12] Weber R.S., Berkey B.A., Forastiere A., Cooper J., Maor M., Goepfert H., Morrison W., Glisson B., Trotti A., Ridge J.A. (2003). Outcome of salvage total laryngectomy following organ preservation therapy: The radiation therapy oncology group trial 91–11. Arch. Otolaryngol. Head Neck Surg..

[13] Stöckli S.J., Pawlik A.B., Lipp M., Huber A., Schmid S. (2000). Salvage surgery after failure of nonsurgical therapy for carcinoma of the larynx and hypopharynx. Arch. Otolaryngol. Head Neck Surg..

[14] Röösli C., Studer G., Stöckli S.J. (2010). Salvage treatment for recurrent oropharyngeal squamous cell carcinoma. Head Neck.

[15] Zafereo M.E., Hanasono M.M., Rosenthal D.I., Sturgis E.M., Lewin J.S., Roberts D.B., Weber R.S. (2009). The role of salvage surgery in patients with recurrent squamous cell carcinoma of the oropharynx. Cancer.

[16] Van Weert S., Leemans C.R. (2021). Salvage surgery in head and neck cancer. Oral Dis..

[17] Garneau J.C., Bakst R.L., Miles B.A. (2018). Hypopharyngeal cancer: A state of the art review. Oral Oncol..

[18] Van den Bovenkamp K., Dorgelo B., Noordhuis M.G., van der Laan B.F.A.M., van der Vegt B., Bijl H.P., Roodenburg J.L., van Dijk B.A.C., Oosting S.F., Schuuring E.M.D. (2018). Viable tumor in salvage neck dissections in head and neck cancer: Relation with initial treatment, change of lymph node size and human papillomavirus. Oral Oncol..

[19] Hamoir M., Schmitz S., Suarez C., Strojan P., Hutcheson K.A., Rodrigo J.P., Mendenhall W.M., Simo R., Saba N.F., D’Cruz A.K. (2018). The Current Role of Salvage Surgery in Recurrent Head and Neck Squamous Cell Carcinoma. Cancers.

[20] Mücke T., Wagenpfeil S., Kesting M.R., Hölzle F., Wolff K.D. (2009). Recurrence interval affects survival after local relapse of oral cancer. Oral Oncol..

[21] Liu S.A., Wong Y.K., Lin J.C., Poon C.K., Tung K.C., Tsai W.C. (2007). Impact of recurrence interval on survival of oral cavity squamous cell carcinoma patients after local relapse. Otolaryngol. Head Neck Surg..

[22] Agra I.M., Carvalho A.L., Ulbrich F.S., de Campos O.D., Martins E.P., Magrin J., Kowalski L.P. (2006). Prognostic factors in salvage surgery for recurrent oral and oropharyngeal cancer. Head Neck.

[23] Van den Bovenkamp K., Noordhuis M.G., Oosting S.F., van der Laan B.F.A.M., Roodenburg J.l., Bijl H.P., Halmos G.B., Platt B.E.C. (2017). Clinical outcome of salvage neck dissections in head and neck cancer in relation to initial treatment, extent of surgery and patient factors. Clin. Otolaryngol..

[24] Hermann R.M., Christiansen H., Rodel R.M. (2013). Lymph node positive head and neck carcinoma after curative radiochemotherapy: A long lasting debate on elective post-therapeutic neck dissections comes to a conclusion. Cancer Radiother..

[25] Malone J., Robbins K.T. (2010). Neck dissection after chemoradiation for carcinoma of the upper aerodigestive tract: Indication and complications. Curr. Opin. Otolaryngol. Head Neck Surg..

[26] Schwartz S.R., Yueh B., Maynard C., Daley J., Henderson W., Khuri S.F. (2004). Predictors of wound complications after laryngectomy: A study of over 2000 patients. Otolaryngol. Head Neck Surg..

[27] Nichols A.C., Kneuertz P.J., Deschler D.G., Lin D.T., Emerick K.S., Clark J.R., Busse P.W., Rocco J.W. (2011). Surgical salvage of the oropharynx after failure of organ-sparing therapy. Head Neck.

[28] Taki S., Homma A., Oridate N., Suzuki S., Suzuki F., Sakashita T., Fukuda S. (2010). Salvage surgery for local recurrence after chemoradiotherapy or radiotherapy in hypopharyngeal cancer patients. Eur. Arch. Otorhinolaryngol..

[29] Lin Y.C., Hsiao J.R., Tsai S.T. (2004). Salvage surgery as the primary treatment for recurrent oral squamous cell carcinoma. Oral Oncol..

[30] Taguchi T., Nishimura G., Takahashi M., Shiono O., Komatsu M., Sano D., Yabuki K.I., Arai Y., Yamashita Y., Yamamoto K. (2016). Treatment results and prognostic factors for advanced squamous cell carcinoma of the head and neck treated with salvage surgery after concurrent chemoradiotherapy. Int. J. Clin. Oncol..

[31] Mueller S.A., Mayer C., Bojaxhiu B., Aeberhard C., Schuetz P.H., Stanga Z., Giger R. (2019). Effect of preoperative immunonutrition on complications after salvage surgery in head and neck cancer. J. Otolaryngol. Head Neck Surg..

[32] White H., Ford S., Bush B., Holsinger F.C., Moore E., Ghanem T., Carroll W., Rosenthal E., Sweeny L., Magnuson J.S. (2013). Salvage surgery for recurrent cancers of the oropharynx: Comparing TORS with standard open surgical approaches. JAMA Otolaryngol. Head Neck Surg..

[33] Huber G.F., Matthews T.W., Dort J.C. (2007). Radiation-induced soft tissue sarcomas of the head and neck. J. Otolaryngol. Head Neck Surg..

